# Supplementary material for: The NMDA receptor subunit GluN2D is a potential target for rapid antidepressant action
Source: Nat Commun. 2025 Nov 26;16:10613. doi: 10.1038/s41467-025-66774-w (PMC12660997; doi:10.1038/s41467-025-66774-w)

Supplementary information for

The NMDA receptor subunit GluN2D is a potential target for rapid antidepressant action

Vestring et al.

Includes:

Supplementary Figures 1 - 7

Supplementary Figure 1

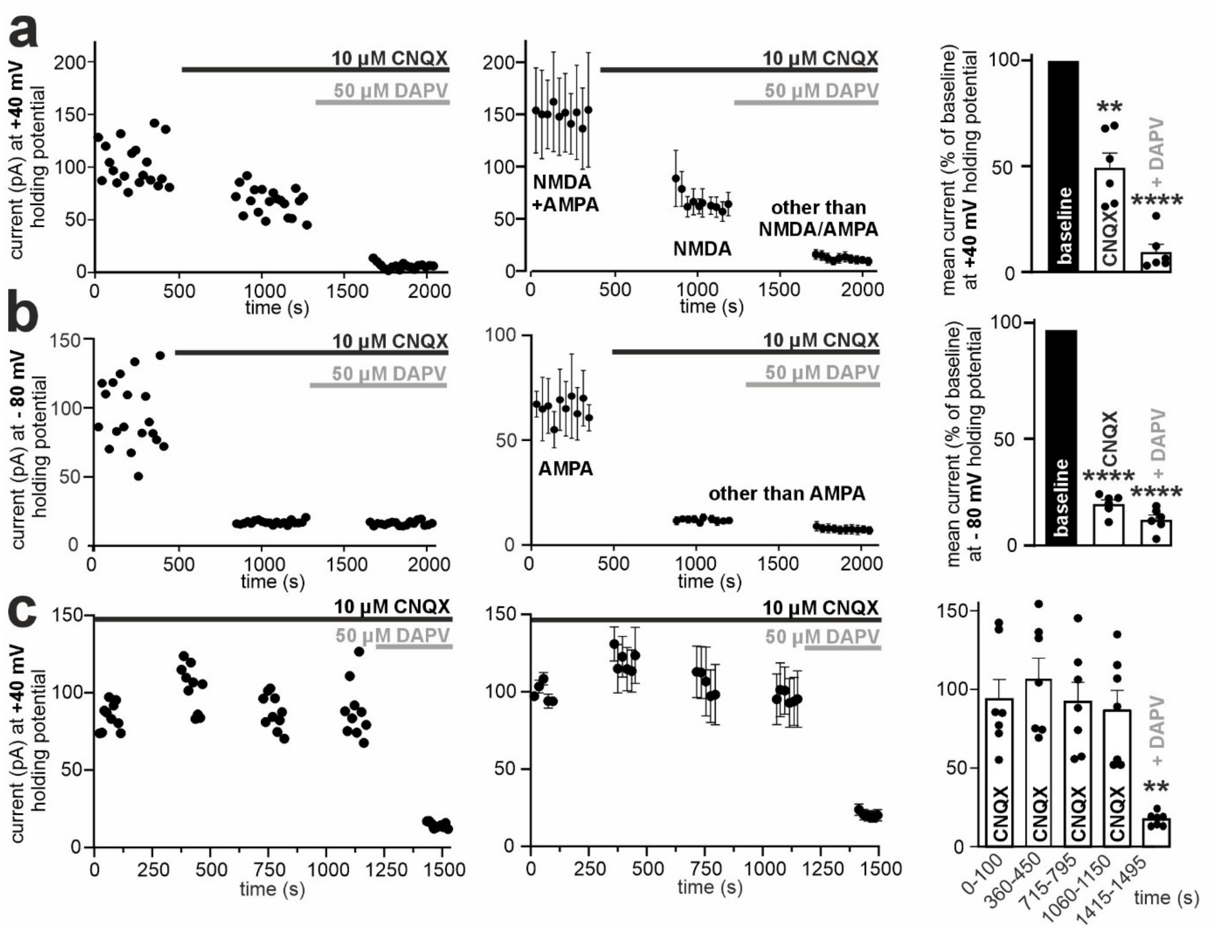

Supplementary Fig. 1: Characterization of AMPAR and NMDA currents using CNQX and DAPV.

a, Representative recordings (maximum current amplitudes; left), averaged time courses (middle) and group analyses (right) of NMDAR currents at +40 mV in CA1 PCs before and after bath application of CNQX (10 µM) to block AMPAR currents and subsequently DAPV (50 µM) to block NMDAR currents. Repeated measures ANOVA, F(141.8), p<0.0001, n=6. Dunnett's multiple comparisons test (DMCT), baseline vs. CNQX: p=0.0013, n=6; baseline vs. CNQX + DAPV: p<0.0001.

b, Representative single-cell recording (left) averaged time courses (middle) and group analyses (right) of NMDAR currents at -80 mV in CA1 PCs before and after bath application of CNQX (10 µM) to block AMPAR currents and subsequently DAPV (50 µM) to block NMDAR currents. Repeated measures ANOVA, F(1308), p<0.0001, n=6. DMCT, baseline vs. CNQX; p<0.0001, n=6; and baseline vs. CNQX + DAPV: p<0.0001, n=6.

c, Representative experiment (left), averaged time course (middle) and group analysis (right) of currents in PCs at +40 mV in the continuous presence of 10 µM CNQX, with DAPV (50 µM) added at the end of the experiments. Repeated measures ANOVA, F(20.06), p<0.0001, n=7). DMCT, baseline vs. DAPV: p=0.0035, n=7.

Data are means ± SEMs. **p<0.01; ****p<0.0001. n=number of cells. Source data are provided as a Source Data file.

Supplementary Figure 2

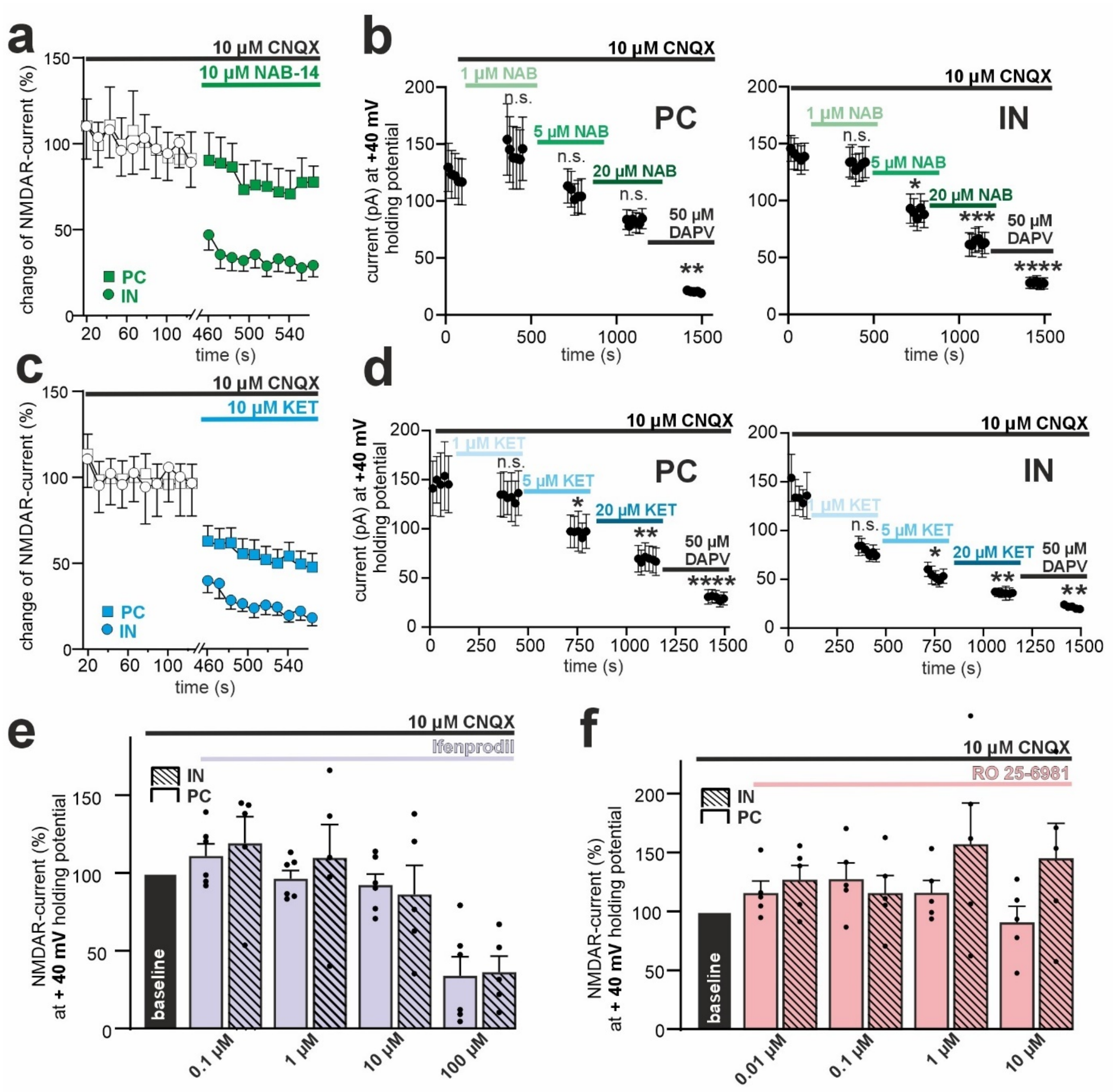

Supplementary Fig. 2: Predominant inhibition of NMDA EPSCs by GluN2D but not GluN2B modulators in interneurons.

a, NMDAR-EPSCs in the presence of 10 µM NAB-14 and CNQX in interneurons (IN: circles) and in pyramidal cells (PCs: squares).

b, NMDAR-EPSCs in PCs (middle) and INs (right) before and after bath application of incremental concentrations (1, 5 and 20 µM) of NAB-14 and DAPV (50 µM) in presence of CNQX (10 µM). PC: Repeated measures ANOVA (RM-ANOVA), F(15.10), p=0.0006, n=11. Post-hoc Dunnett's multiple comparisons test(DMCT): baseline vs. D-APV: p=0.0021. IN: RM-ANOVA, F(35.06), p<0.0001, n=12. DMCT, baseline vs. 1 µM NAB-14: p=0.731, vs. 5 µM NAB-14: p=0.020, vs. 20 µM: p=0.0003, vs. DAPV: p<0.0001.

c, Reduction in NMDAR-EPSCs induced by 10 µM KET in INs (circles) and in PCs (squares) in the presence of 10 µM CNQX.

d, NMDAR-EPSCs in the presence of CNQX (10 µM) in PCs (middle) and INs (right) before and after bath application of incremental concentrations (1, 5 and 20 µM) of KET and DAPV (50 µM). PC: Friedman test, Friedman statistic=37.04, $p<0.0001$, n=10. DMCT baseline vs. 5 µM KET: p=0.0288, vs. 20 µM KET: p=0.0016, vs. DAPV: $p<0.0001$. IN: RM-ANOVA, F(23.69), p=0.0009, n=7. DMCT baseline vs. 5 µM KET: p=0.0225, vs. 20 µM: p=0.0071, vs. DAPV: p=0.0024.

e, NMDAR-EPSCs in PC and IN during increasing Ifenprodil concentrations (0.1–100 µM), D-APV (50 µM) and CNQX(10µM). Two-way RM-ANOVA showed a main effect of concentration (F[27.10], $p<0.0001$), but no effect of cell type (F[0.13], p=0.72) and no interaction (F[0.35], p=0.79). Šídák's post-hoc test revealed no differences between PCs (n=6) and INs (n=5).

f, NMDAR-EPSC in PCs and INs during bath application of increasing RO 25-6981 concentrations (0.01- 10 µM), D-APV (50 µM) and CNQX(10 µM). Two-way RM-ANOVA revealed a concentration × cell type interaction (F[4.03], p=0.019), but no main effects of concentration (F[1.24], p=0.319) or cell type (F[0.93], p=0.364). Šídák's post-hoc tests detected no significant differences between PCs (n=5) and INs (n=5).

The data are presented as the means ± SEMs. *$p<0.05$, **$p<0.01$, ***$p<0.001$, ****$p<0.0001$. n=number of cells. Source data are provided as a Source Data file.

Supplementary Figure 3

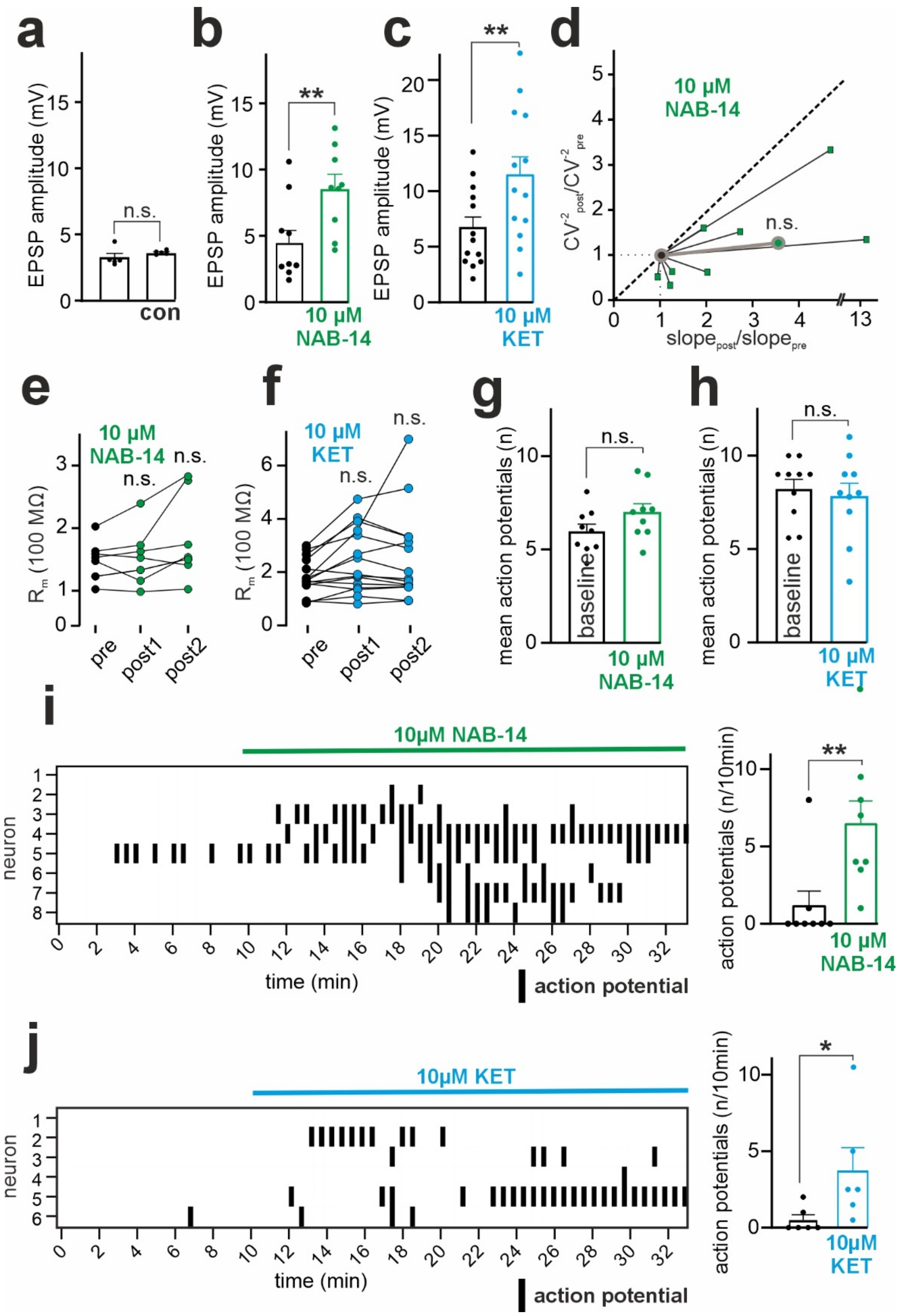

Supplementary Fig. 3: NAB-14 and KET increase EPSPs via postsynaptic inhibition of hippocampal microcircuits.

a, Mean EPSP amplitudes in the MICA remained stable over 30 min in the control condition. Two-tailed paired t-test (p=0.507, n=5).

b, Mean EPSP amplitudes in the MICA increased after the addition of 10 µM NAB-14 to the bathing solution. Two-tailed paired t- test (p=0.0019, n=9).

c, Mean EPSP amplitudes in the MICA increased after the addition of 10 µM KET to the bathing solution. Two-tailed paired t-test (p=0.0019, n=13).

d, CV analysis of the effect of NAB-14 wash-in during MICA, consistent with a postsynaptic mechanism. The gray circles represent the averages of all analyzed cells. One-sample two-tailed t-test against mean of 1 (p=0.6158, n=8).

e, PC input resistance (R_m) did not change at either 10, 20 or 30 min in the MICA after NAB-14 (10 µM) bath application. Repeated measures ANOVA, F=3.367, p=0.101, n=7.

f, PC input resistance (R_m) did not change at either 10, 20 or 30 min in the MICA after KET (10 µM) bath application. Repeated measures ANOVA, F=3.884, p=0.057, n=15.

g, No significant changes in the number of APs during the burst of APs in the MICA protocol after bath application of 10 µM NAB-14. Two-tailed paired t-test (p=0.877, n=9).

h, No significant changes in the number of APs during the burst of APs in the MICA protocol after bath application of 10 µM KET. Two-tailed paired t-test (p=0.4633, n=10).

i, Raster blot (left) and group analysis (right) showing that NAB-14 (10 µM) bath application leads to the conversion of subthreshold EPSPs to APs. Two-tailed Wilcoxen test (p=0.0078, n=8).

j, Raster blot (left) and group analysis (right) showing that KET (10 µM) bath application leads to the conversion of subthreshold EPSPs to APs. Two-tailed Wilcoxen t-test (p=0.0313, n=6).

The data are presented as the means ± SEMs. *p<0.05, **p<0.01. n=number of cells. Source data are provided as a Source Data file.

Supplementary Figure 4

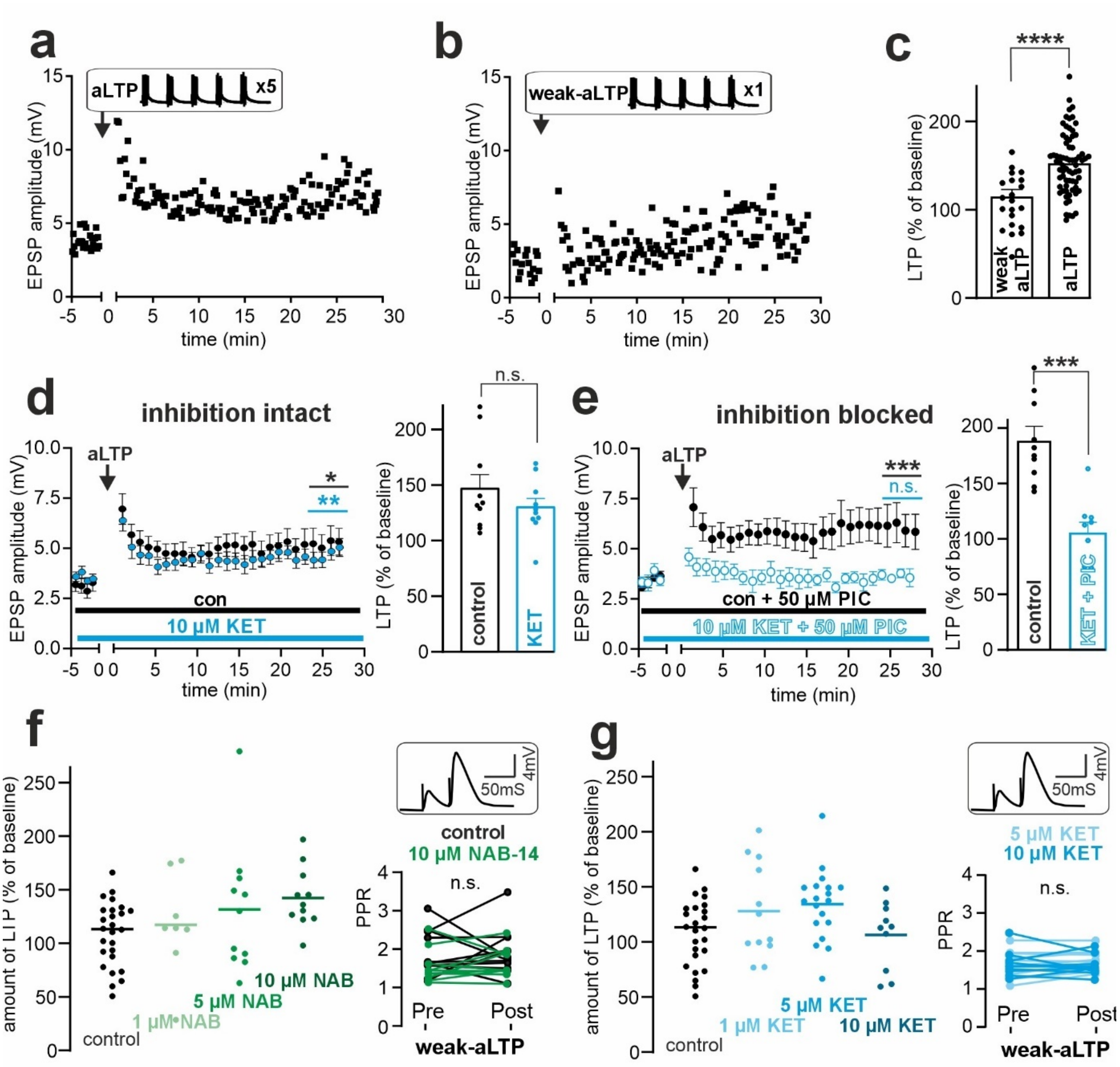

Supplementary Fig. 4: Interneurons shape hippocampal LTP.

a, Representative aLTP experiment. Each square represents the maximum EPSP amplitude before and after 125 EPSP→AP pairings (inset).

b, Representative weak associative LTP (weak-aLTP) recording with 25 EPSP→AP pairings (inset).

c, Significant LTP was induced by both protocols but was significantly greater in the aLTP group than in the weak-aLTP group. Two-tailed unpaired t-test ($p<0.0001$, n=25 [weak-aLTP], n=70 [aLTP]).

d, (left) Time scale of aLTP in control solution (black) or in the presence of 10 µM KET (blue) showing a significant increase of EPSPs after LTP induction (25-30min.) compared to baseline (0-5min). Two tailed paired t-tests: KET: $p=0.0037$, Control: $p=0.01$. (right) Group analysis revealed no difference between the conditions. Mann-Whitney test ($p=0.58$) n=10 for both groups.

e, Experimental setup identical to that in (d) but in the presence of the $GABA_A$ antagonist picrotoxin (PIC, 50 µM) both under control conditions (black) and together with KET (blue) Two tailed Wilcoxen

test Control: p=0.002, Two tailed paired t-test KET: p=0.8428. Group comparison: Two-tailed unpaired t-test (p<0.0005) control n=10; KET n=8).

f, (Left) Single-experiment overview of weak-aLTP experiments analyzed in Fig. 4a and (right) analysis of paired-pulse ratio (PPR) pre- vs. post-weak-aLTP induction in the presence of 10 µM NAB-14 in the bathing solution (green) and under control conditions (black, inset: representative paired-pulse EPSP trace). Two-tailed paired t-tests: control: p=0.83, n=12; NAB-14: p=0.48, n=11.

g, (Left) Single-experiment overview of weak-aLTP experiments analyzed in Fig. 3c. (Right) analysis of PPR pre- vs. post-weak-aLTP induction in the presence of 5 or 10 µM KET in the bathing solution. Two-tailed paired t-tests: 5 KET: p=0.15, n=9; 10 KET: p=0.56, n=10.

The data are presented as the means ± SEMs. *p<0.05, **p<0.01, ****p<0.0001. n=number of cells. Source data are provided as a Source Data file.

Supplementary Figure 5

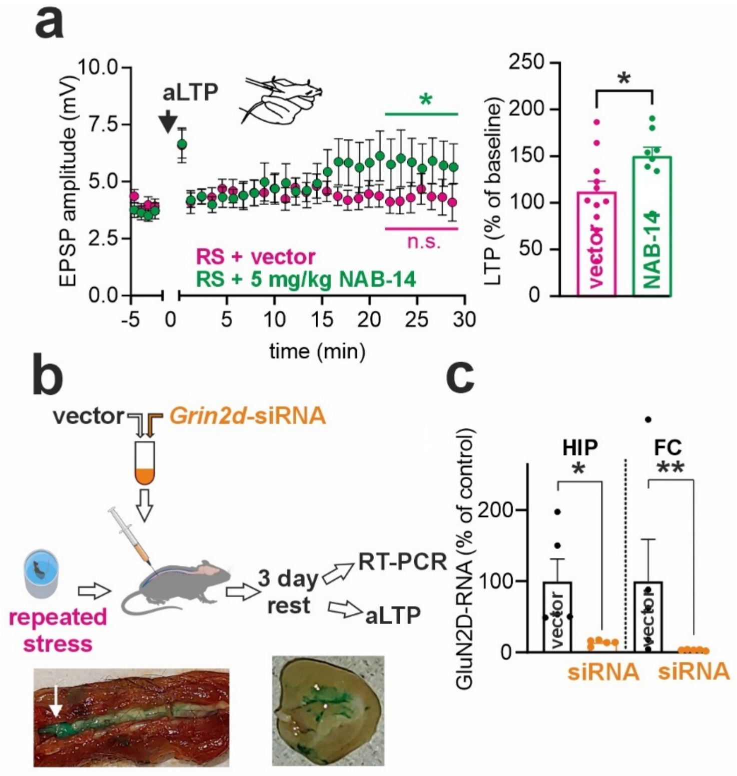

Supplementary Fig. 5: 5 µM NAB-14 partially restores LTP after CDM; GluN2D siRNA injection effectively reduces GluN2D RNA levels.

a, (Left, time course) A single intraperitoneal NAB-14 injection (5 mg/kg, green; two-tailed paired t-test baseline vs. 20-30 min, p=0.0345, n=9) resulted in a partial rescue of aLTP in RS mice compared to that in RS mice that received an injection of the vector used to dissolve NAB-14 (magenta; two-tailed paired t-test baseline vs. 20-30 min, p=0.4354, n=11) (Right, group analysis) Two-tailed unpaired t-test, p=0.044, vector n=11; NAB-12 n=9. n=number of cells.

b, Schematic overview of the siRNA protocol. *Grin2d* siRNA was injected intrathecally after exposure to RS, followed by three days of rest. Thereafter, readout measurements, real-time PCR and *ex vivo* aLTP measurements were performed. Green ink was injected in some control experiments into anesthetized mice between the L5 and L6 vertebrae (white arrow) to confirm the accuracy and reliability of the intrathecal injection technique. The ink diffused throughout the vertebral canal (top) and into the brain (bottom, coronal section).

c, RT-PCR showed that GluN2D mRNA expression was significantly lower in the hippocampus (HIP) and the frontal cortex (FC) after siRNA treatment than after vector injection. HIP: two-tailed unpaired t-test, p=0.0244, n=5. FC: Mann Whitney test, p=0.008, n=5. n=number of animals.

The data are presented as the means ± SEMs. *p<0.05, **p<0.01. Source data are provided as a Source Data file. Created in BioRender. Vestring, S. (2025) https://BioRender.com/dpm4zwz.

Supplementary Figure 6

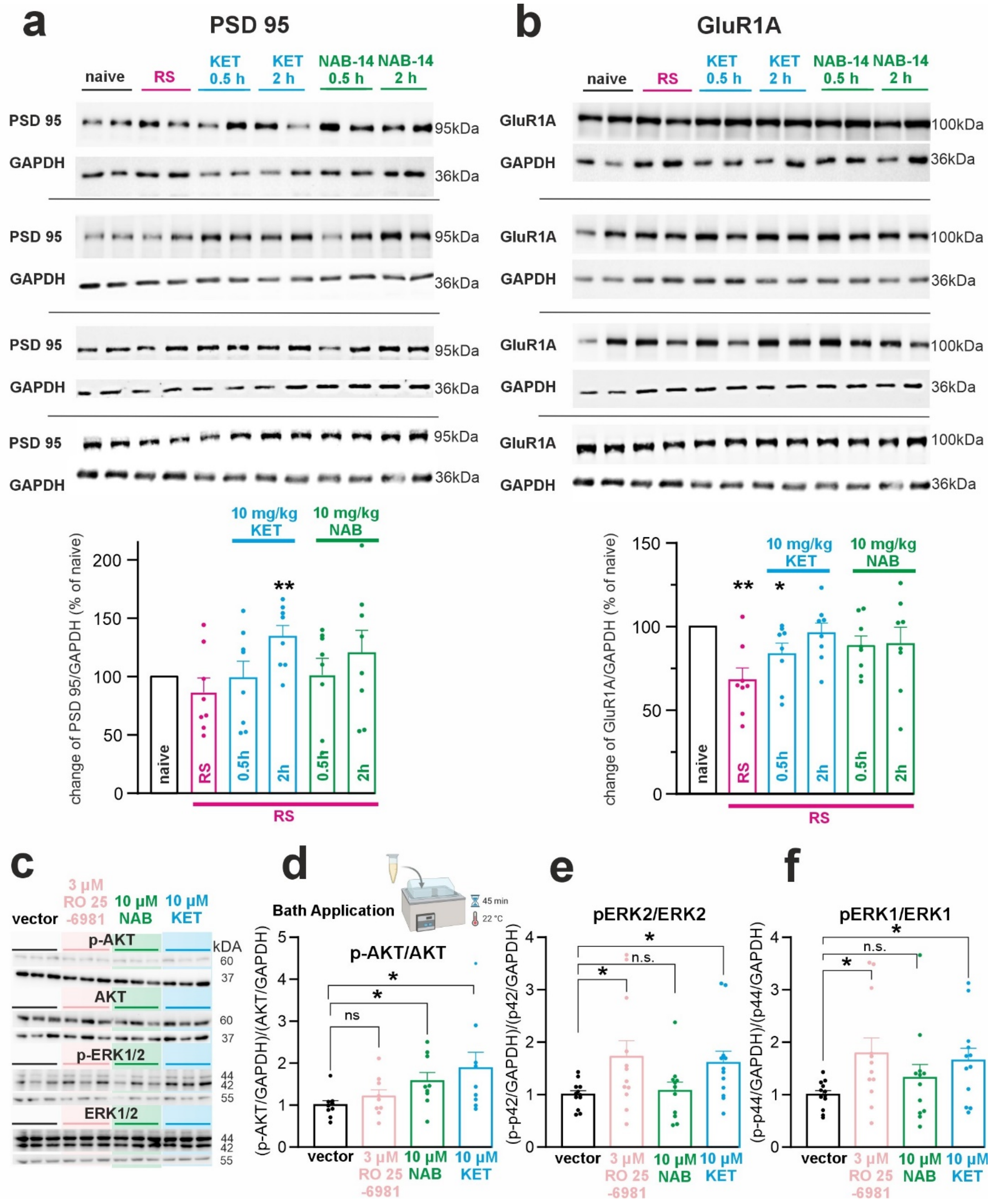

Supplementary Fig. 6: Western blots of PSD 95, GluR1A, AKT and ERK1/2.

a, Western blot (WB) traces (top) and quantification (bottom) of PSD-95 protein levels in four experimental groups: naïve, repeated stress (RS), RS+KET (10mg/kg; 30min and 2h), and RS+NAB-14 (10mg/kg; 30min and 2h), normalized to the naïve group. Two-tailed one-sample t-tests vs. naïve: RS: p=0.306, KET 30min: p=0.929, KET 2h: p=0.0098, NAB-14 30min: p=0.933, NAB-14 2h: 0.344; all n=8.

b, WB-traces (top) and quantification (bottom) of GluR1A protein levels in the same groups shown in (a), normalized to naïve animals to illustrate stress- and treatment induced regulation. Two-tailed

one-sample t-tests vs. naive: RS: p=0.003, KET 30min: p=0.041, KET 2h: p=0.543, NAB-14 30min: p=0.095, NAB-14 2h: 0.335; all n=8. GAPDH (36 kDa) served as loading control from the same experiments parallel processed blots.

c, Representative WB traces for p-Akt/Akt, pERK1/ERK1, and pERK2/ERK2 following 45-minute bath application of saline (vehicle), RO 25-6981 (3 µM), NAB-14 (10 µM), or KET (10 µM) to acute brain slices. Loading control (Tubulin, 55 kDa) was blotted on the same membrane except for p-ERK1/2, where it derived from the same experiment (parallel processing of gel and blot).

d, Quantification of p-AKT/AKT ratios. Kruskal-Wallis test revealed a significant difference among groups (H(3)=8.418, p=0.0381, all n=9). Dunn's post hoc test showed significant increases in p-AKT levels in NAB-14 (p=0.015) and KET groups (p=0.015) compared to saline. RO 25-6981 did not differ from saline (p=0.314).

e, Quantification of pERK2/ERK2 (p-p42/p42) ratios. Kruskal-Wallis analysis showed a significant group effect (H(3)=7.964, p=0.0468). Dunn's post hoc test revealed that both RO 25-6981 (p=0.034) and KET (p=0.027) increased pERK2 levels relative to saline. NAB-14 showed no effect (p=0.729). All groups n=12.

f, Quantification of pERK1/ERK1 (p-p44/p44) ratios. Kruskal-Wallis analysis showed a difference across groups (H(3)=8.211, p=0.042). Dunn's post hoc testing indicated increased pERK1 in RO 25-6981 (p=0.017) and KET (p=0.018) compared to saline. NAB-14 had no effect (p=0.355). All groups n=12.

The data are presented as the means ±SEMs. *p<0.05, **p<0.01. n=number of samples. Source data are provided as a Source Data file. Created in BioRender. Vestring, S. (2025) https://BioRender.com/dpm4zwz.

Supplementary Figure 7

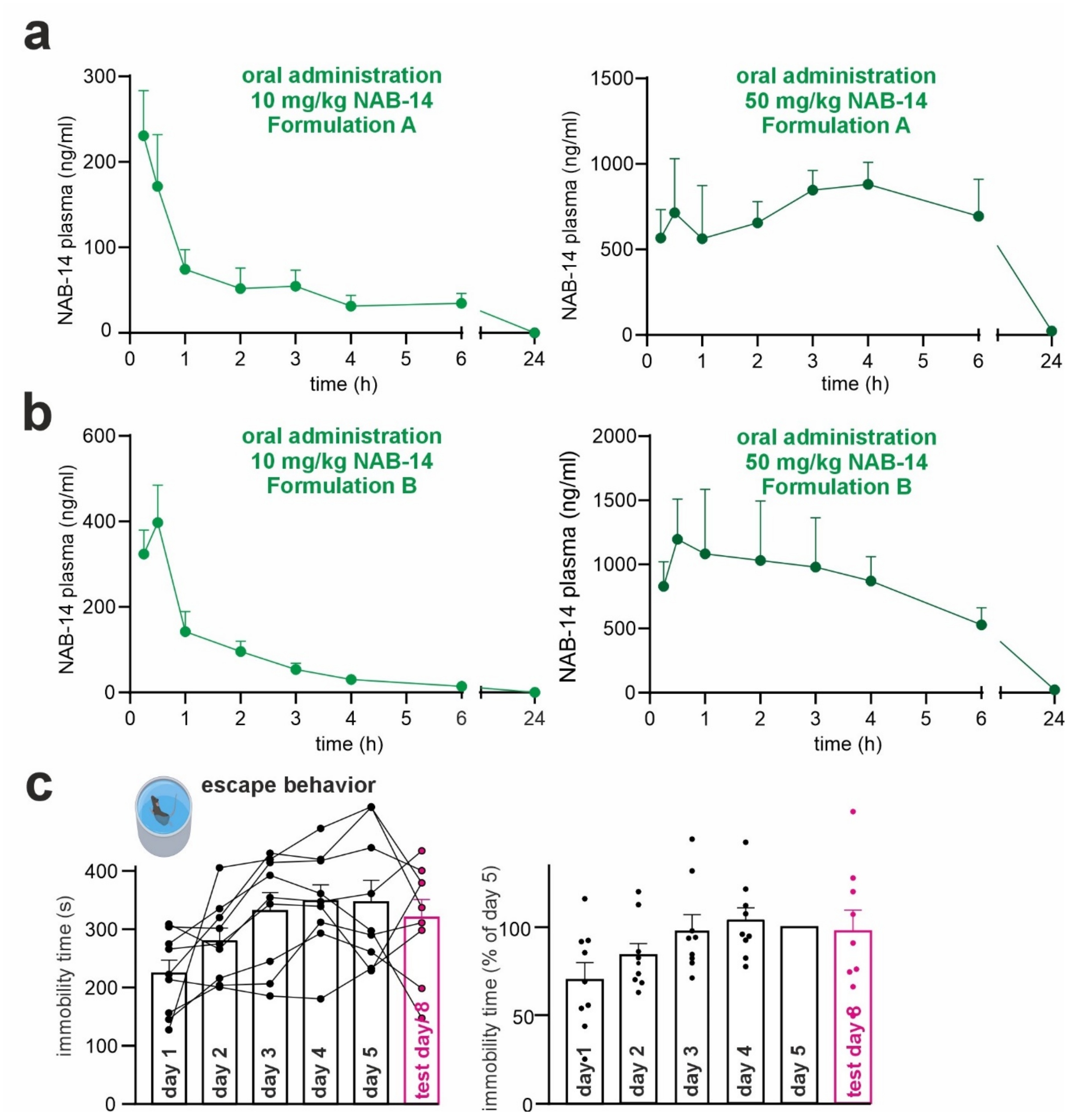

Supplementary Fig. 7: Pharmacokinetic profiles after oral administration of NAB-14 and time course of immobility time in the RS protocol under control conditions.

a, Plasma concentrations of NAB-14 were measured following oral administration of 10 mg/kg (left) and 50 mg/kg (right) of Formulation A (see Methods). The curve represents the mean plasma concentration over time ± SEM (n=3 male rats per dose and time point).

b, Pharmacokinetic profiles of NAB-14 after oral dosing with 10 mg/kg (left) and 50 mg/kg (right) of Formulation B. Time-dependent plasma levels indicate formulation-dependent absorption. (n=3 male rats per group and time point).

c, Averaged immobility times in the RS protocol gradually increased from day 1 to day 5 and remained stable on the test day 8. (Left): absolute values and time course of single experiments; (right): values normalized to day 5, n=9.

The data are presented as the means ± SEMs. n=number of animals. Source data are provided as a Source Data file. Vestring, S. (2025) https://BioRender.com/dpm4zwz.

Extended Source Data

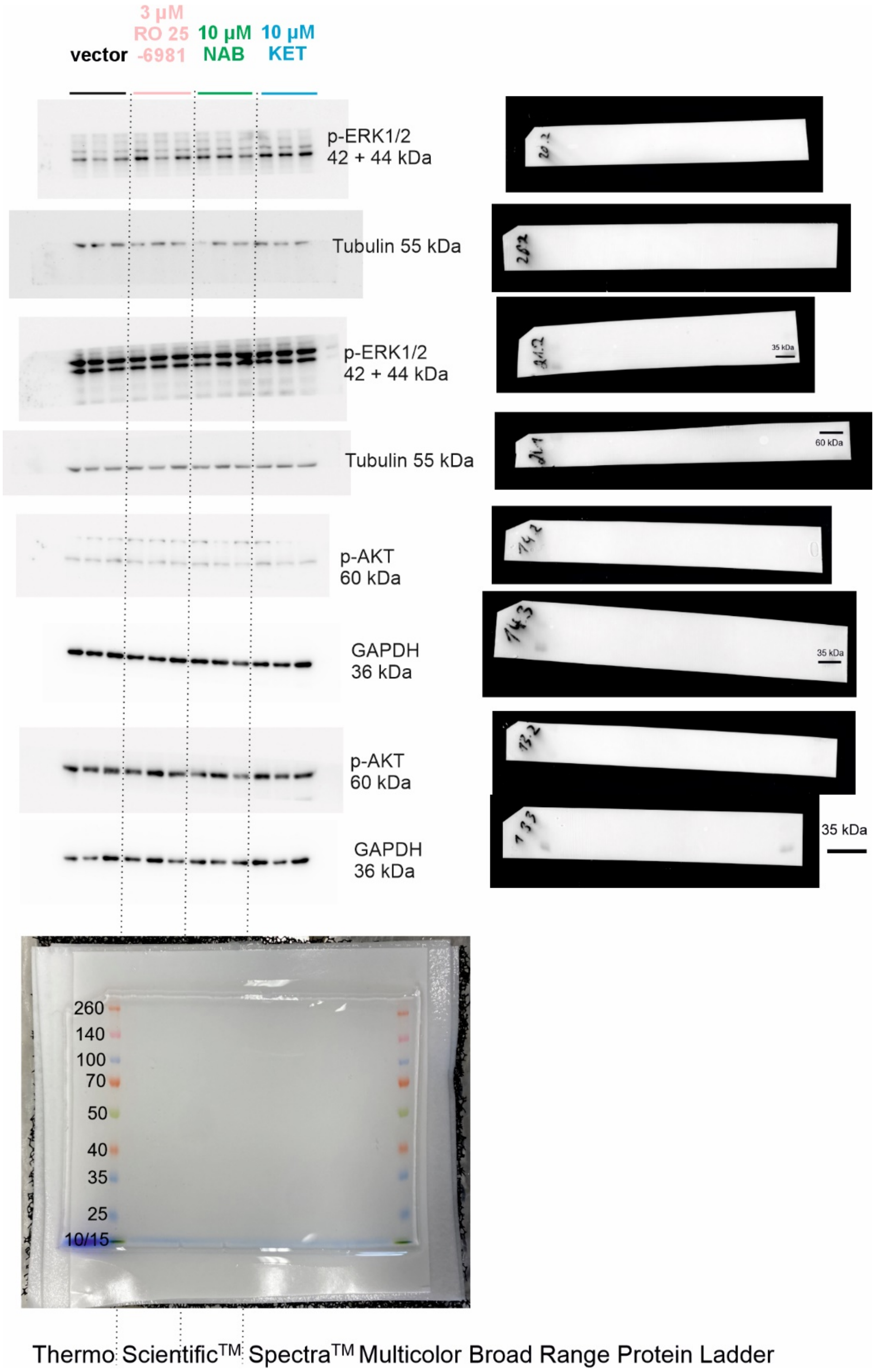

Supplement: Supplementary file 1 — Supplementary Information [file 41467_2025_66774_MOESM1_ESM.pdf]
