# Supplementary material for: The NMDA receptor subunit GluN2D is a potential target for rapid antidepressant action
Source: Nat Commun. 2025 Nov 26;16:10613. doi: 10.1038/s41467-025-66774-w (PMC12660997; doi:10.1038/s41467-025-66774-w)
Supplement: Supplementary file 2 — Reporting Summary [file 41467_2025_66774_MOESM2_ESM.pdf]

nature portfolio

Corresponding author(s): Claus Normann

Last updated by author(s): 10.10.2025

Reporting Summary

Nature Portfolio wishes to improve the reproducibility of the work that we publish. This form provides structure for consistency and transparency in reporting. For further information on Nature Portfolio policies, see our Editorial Policies and the Editorial Policy Checklist.

Statistics

For all statistical analyses, confirm that the following items are present in the figure legend, table legend, main text, or Methods section.

n/a	Confirmed	
☐	☒	The exact sample size (n) for each experimental group/condition, given as a discrete number and unit of measurement
☐	☒	A statement on whether measurements were taken from distinct samples or whether the same sample was measured repeatedly
☐	☒	The statistical test(s) used AND whether they are one- or two-sided<br>*Only common tests should be described solely by name; describe more complex techniques in the Methods section.*
☒	☐	A description of all covariates tested
☐	☒	A description of any assumptions or corrections, such as tests of normality and adjustment for multiple comparisons
☐	☒	A full description of the statistical parameters including central tendency (e.g. means) or other basic estimates (e.g. regression coefficient) AND variation (e.g. standard deviation) or associated estimates of uncertainty (e.g. confidence intervals)
☐	☒	For null hypothesis testing, the test statistic (e.g. F, t, r) with confidence intervals, effect sizes, degrees of freedom and P value noted<br>*Give P values as exact values whenever suitable.*
☒	☐	For Bayesian analysis, information on the choice of priors and Markov chain Monte Carlo settings
☒	☐	For hierarchical and complex designs, identification of the appropriate level for tests and full reporting of outcomes
☒	☐	Estimates of effect sizes (e.g. Cohen's d, Pearson's r), indicating how they were calculated

Our web collection on statistics for biologists contains articles on many of the points above.

Software and code

Policy information about availability of computer code

Data collection	Electrophysiology: Patchmaster NEXT 1.2, HEKA. Behavior: IntelliCage Plus software, TSE Systems; EthoVision XT software, Noldus, Morphology: Fiji, 3D Weka Segmentation plugin, Imaris 10.0, Oxford Instruments. Microscopy images were acquired using ZEN Black software (Carl Zeiss). Molecular modelling: PrepWizard/Glide/OPLS4 force field/LigPrep (Schrödinger Software Suite 2021-4). Fiberphotometry data were collected using LabView (National Instruments).
Data analysis	Electrophysiology: Patchmaster & Fitmaster NEXT 1.2., HEKA. Immunobloting: ImageJ 1.53s (https://imagej.nih.gov/ij/). Morphology: Imaris 10.0, Oxford Instruments. Behaviour during fiberphotometry was tracked using Deeplabcut. Fiberphotometry data were analyzed with custom script writen in MATLAB (Mathworks) and Python. All statistical analyses: GraphPad Prism 8.3.0, GraphPad Software

For manuscripts utilizing custom algorithms or software that are central to the research but not yet described in published literature, software must be made available to editors and reviewers. We strongly encourage code deposition in a community repository (e.g. GitHub). See the Nature Portfolio guidelines for submitting code & software for further information.

Data

Policy information about availability of data

All manuscripts must include a data availability statement. This statement should provide the following information, where applicable:

- Accession codes, unique identifiers, or web links for publicly available datasets
- A description of any restrictions on data availability
- For clinical datasets or third party data, please ensure that the statement adheres to our policy

The electrophysiological, imaging, pharmacokinetic, and behavioral data generated in this study have been deposited in the Figshare database under accession code dx.doi.org/10.6084/m9.figshare.30436471. The Supplementary data are available in the Figshare database under accession code dx.doi.org/10.6084/m9.figshare.30436492. Source data is available in Source data table. All code is available including a how to use readme description under dx.doi.org/10.6084/m9.figshare.29245961

Research involving human participants, their data, or biological material

Policy information about studies with human participants or human data. See also policy information about sex, gender (identity/presentation), and sexual orientation and race, ethnicity and racism.

Reporting on sex and gender	NA
Reporting on race, ethnicity, or other socially relevant groupings	NA
Population characteristics	NA
Recruitment	NA
Ethics oversight	NA

Note that full information on the approval of the study protocol must also be provided in the manuscript.

Field-specific reporting

Please select the one below that is the best fit for your research. If you are not sure, read the appropriate sections before making your selection.

[x] Life sciences [ ] Behavioural & social sciences [ ] Ecological, evolutionary & environmental sciences

For a reference copy of the document with all sections, see nature.com/documents/nr-reporting-summary-flat.pdf

Life sciences study design

All studies must disclose on these points even when the disclosure is negative.

Sample size	Group size estimates were based on previous experience with the experimental design and the mouse models. For behavioral experiments, a formal power calculation was done with the Institute of Medical Biometry and Medical Informatics, University of Freiburg (n=10 per group for most experimental series). For Western blot and RT-PCR, IHC and fiberphotometry we aimed for a group size of 4-6, for electrophysiological experiments and morphologic analysis 7-10. In several cases, especially for baseline measurements, the group sizes exceeded these numbers, since several batches of mice were tested from independent experimentators and pooled for final analyses.
Data exclusions	For electrophysiological recordings, clear exclusion criteria were set, which are described in the Methods. For other assessments, no data were excluded.
Replication	For electrophysiological recordings, key experiments and baseline assessments were replicated by different experimentators. A maximum of two hippocampal slices per animal was used. All groups included at least 4 animals with equal sex ratios. All other experiments were replicated at least 3 times and all attempts were successful.
Randomization	The animals were age-matched, no further randomization was used.
Blinding	In all applicable experiments, blinding was implemented to minimize bias during both data collection and analysis. In behavioral studies, experiments were either videotaped or automatically tracked. Behavioral scoring was conducted offline by researchers blinded to the treatment conditions, using coded videos without identifying information.For Western blot, RT-PCR, and dendritic spine morphology experiments, samples were labeled with anonymized codes prior to processing. Data acquisition followed standard protocols, and all quantification and analysis were performed by researchers blinded to the group identities.In electrophysiological and fiber photometry experiments, experimenters conducting the recordings were blinded to treatment or experimental condition through the use of coded sample IDs. Subsequent analysis was also performed with group assignments concealed.Pharmacokinetic studies were not blinded due to the procedural requirements of sample collection and processing, which necessitated knowledge of the treatment conditions.

Reporting for specific materials, systems and methods

We require information from authors about some types of materials, experimental systems and methods used in many studies. Here, indicate whether each material, system or method listed is relevant to your study. If you are not sure if a list item applies to your research, read the appropriate section before selecting a response.

Materials & experimental systems

n/a	Involved in the study
☐	☒ Antibodies
☒	☐ Eukaryotic cell lines
☒	☐ Palaeontology and archaeology
☐	☒ Animals and other organisms
☒	☐ Clinical data
☒	☐ Dual use research of concern
☒	☐ Plants

Methods

n/a	Involved in the study
☒	☐ ChIP-seq
☒	☐ Flow cytometry
☒	☐ MRI-based neuroimaging

Antibodies

Antibodies used

Anti-VGLUT1 (guinea pig polyclonal) Millipore Cat no: AB5905
Lot Number: 3878831
RRID:AB_2301751
1:5.000

Anti-GAD65 (mouse) Abcam Cat no: ab26113
Lot Number:
GR3308495-2
RRID:AB_448989
1:500

Anti Phospho-p44/42 MAPK (Erk1/2) antibody Cell Signaling Technology Cat no: 9101
Lot Number: 31
RRID: AB_331646
1:10.000

Secondary antibody goat polyclonal anti-guinea pig Alexa
Fluor 594
Invitrogen Cat no: A-11076
Lot Number: 2540867
RRID:AB_2534120
2 µg/mL

Secondary antibody goat polyclonal anti-mouse Alexa Fluor
488
Invitrogen Cat no: A-32723
Lot Number: XA336883
RRID:AB_2633275
2 µg/mL

Anti-GluA1-NT (mouse) Millipore Cat no: MAB2263
Lot Number: 3847897
RRID:AB_11212678
1:2000

Anti-PSD95 (rabbit) Cell Signaling Cat no: 2507
Lot Number: 4
RRID:AB_561221
1:2000

Anti-GAPDH (mouse) Abcam Cat no: ab8245
Lot Number: 1035914-6
RRID:AB_2107448
1:10.000

Secondary antibody anti-mouse (sheep) GE Healthcare Cat no: NA931

Lot Number:
17556470/18111935
RRID:AB_772210
1:20.000

Anti p44/42 MAPK (ERK1/2) antibody Cell Signaling Technology Cat no: 9102
Lot Number: 30
RRID: AB_330744
1:10.000

Anti Phospho-p44/42 MAPK (Erk1/2) antibody Cell Signaling Technology Cat no: 4376
Lot Number: 21
RRID: AB_331772
1:10.000

Anti beta-Tubulin antibody Sigma Aldrich Cat no: T4026
Lot Number: 0000324939
RRID: AB_477577
1:2.000

Anti Akt Antibody Cell Signaling Technology Cat no: 9272
Lot Number: 30
RRID: AB_329827
1:1.000

Anti Phospho-Akt antibody Cell Signaling Technology Cat no: 4060
Lot Number: 27
RRID: AB_2315049
1:2.000

Anti-GAPDH antibody Cell Signaling Technology Cat no: 2118
Lot Number: 16
Clone: 14C10
RRID: AB_561053
1:1.000

Anti-rabbit antibody SeraCare Cat no: 5450-0010
Lot Number: 10571726
RRID: AB_3075498
1:2000

Validation

Anti VGLUT1 (guinea pig polyclonal): Validated by Milipore and used in multiple studies as per company's website (ie. Turner et al., 2015, Nature;. Brigidi et al., 2015, Nat Commun).
Anti-GAD65 (mouse): Validated by abcam and used in multiple studies as per company's website (ie. Chai G et al., 2021, Neuron; Kang W et al., 2022, Nat. communications).
Secondary antibody anti-guinea pig Alexa Fluor 594: Validated by Invitrogen and used in multiple studies as per company's website in Stillman et al., 2023, Nat. Communications; Chen et al., 2023, IScience).
Secondary antibody goat polyclonal anti-mouse Alexa Fluor 488: Validated by Invitrogen and used in multiple studies as per company's website in Monteil et al., 2023, Nat. Communications; Zheng et al., 2023, Nature Structural Molecular Biology).
Anti-GluA1-NT (mouse): Validated by Milipore and used in multiple studies as per company's website (ie. Mignogna et al., 2015, Nat. communications;. Sephton et al., 2014, PNAS).
Anti-PSD95 (rabbit): Validated by Cell Signaling and used in multiple studies as per company's website (ie. Wei-Dong et al., 2004, Neuron;. Chetkovich et al., 2002, J Neurosci.).
Anti-GAPDH (mouse): Validated by Abcam and used in multiple studies as per company's website (ie. Giannaki et al., 2022, J Cell Physiol; Meng et al., 2022, J Cancer).
Secondary antibody anti-mouse (sheep): Validated by GE Healthcare and used in multiple studies as per company's website (ie. Yifan et al., 2020, eLife; De Backer et al., 2021, Sci. reports).
Secondary antibody anti-rabbit (donkey): Validated by GE Healthcare and used in multiple studies as per company's website (ie. Clemente et al., 2022, Nat. communications; Hou et al., 2021, Nat. communications).
Anti p44/42 MAPK (ERK1/2) antibody Cell Signaling Technology: Validated by company and used in multiple studies as per company's website (ie. Roux et al., 2004, Microbiol Mol Biol Rev and Baccarini, M.,2005, FEBS Lett, and Meloche et al., 2007, Oncogene)
Anti Phospho-p44/42 MAPK (Erk1/2) antibody Cell Signaling Technology: Validated by company and used in multiple studies as per company's website (ie. Kortenjann et al.,1994, Mol Cell Biol. And Owens et al., 2007, Oncogene.)
Anti beta-Tubulin antibody Sigma Aldrich: Validated by company and used in multiple studies as per company's website (ie. Rouleau et al., 2011, Stem Cells, and Surendran et al. 2005 Journal of the American Society of Nephrology.)
Anti Akt Antibody Cell Signaling Technology: Validated by company and used in multiple studies as per company's website (ie. Franke et al., 1997, Cell and Burgering et al., 1995 Nature.)
Anti Phospho-Akt antibody Cell Signaling Technology: Validated by company and used in multiple studies as per company's website (ie. Franke et al., 1997, Cell and Burgering et al., 1995 Nature.)

Anti-GAPDH antibody Cell Signaling Technology: Validated by company and used in multiple studies as per company's website (ie. Zheng et al., 2003, Cell and Bae et al., 2006, Proc. Natl. Acad. Sci.)
Anti-rabbit antibody SeraCare: Validated by company as per company's website (SeraCare KPL Cat# 5450-0010, RRID:AB_3075498)
Anti Phospho-p44/42 MAPK (Erk1/2) antibody Cell Signaling: alidated by company and used in multiple studies as per company`s website (i.e.Owens, D.M. and Keyse, S.M., 2007. Oncogene, Marais, R. et al., 1993, Cell)

Animals and other research organisms

Policy information about studies involving animals; ARRIVE guidelines recommended for reporting animal research, and Sex and Gender in Research

Laboratory animals	Mice: Wild-type C57Bl6N mice were obtained from Janvier (Le Genest-Saint-Isle, France) and Thy1-GCaMP6 mice from Jackson Laboratory (C57BL/6J-Tg(Thy1-GCaMP6f)GP5.5Dkim/J; Stock No: 024276/GP5.5). SOM-Cre (SST tm2.1(cre)Zjh/J) mice were provided by the Department of Biomedicine Basel and SOM-IRS-Cre/J from the Department of Physiology of the University of Freiburg. Animals were housed at the accredited facilities of the Universities of Freiburg and Strasbourg. Adult mice (10-14 weeks) were group-housed (up to five per cage) under standardized conditions (12 h light/dark cycle, controlled temperature (21°C ±2°) and humidity (55% ± 10%), food and water ad libitum, nesting material and enrichment).<br>All animals were handled by daily tunnel handling for 5 min over five days prior to behavioral testing to reduce stress. Welfare was monitored at least once daily by trained staff and additionally after invasive procedures, with health assessments including body weight, coat condition, locomotor activity, and posture. For stereotactic and other invasive procedures, anesthesia was induced and maintained with isoflurane, and peri-operative analgesia was provided (carprofen, buprenorphine). At the end of experiments, euthanasia was performed according to downstream analysis: for electrophysiology, mice were preoxygenated in 100% O_2 for 5 min before cervical dislocation and decapitation; for histology, mice were deeply anesthetized and transcardially perfused with fixative; for behavioral cohorts not undergoing perfusion or slice preparation, euthanasia was performed by cervical dislocation and decapitation without preoxygenation.<br>Rats: For studies conducted in rats, male Sprague Dawley rats (Crl:CD(SD), 9 weeks old, 250–300 g) were bred and housed at Aptuit facilities in Verona, Italy. Animals were maintained in solid-bottomed plastic cages with sawdust bedding and either external watering systems or water bottles. Two to three rats of the same treatment group were housed per cage under controlled environmental conditions (temperature 20–22 °C; humidity 45–65%; 12 h light/dark cycle with species-appropriate illumination). Rats received a standard maintenance diet (Altromin 1324 IRR, rat diet) and filtered tap water ad libitum.
Wild animals	The study did not involve wild animals.
Reporting on sex	Mice from both sexes with equal sex ratios were used. Sex-based analyses were not performed since the experiments were not powered to assess sex differences.
Field-collected samples	The study did not involve samples collected in the field.
Ethics oversight	Mice: All experimental procedures were approved by the relevant authorities in Germany (Regierungspräsidium Freiburg, TV-G-20-106, TV-G-20-87, TV-G-19-10, TV-G20-141, TV-G21-69, TV-G22-116, TV-G-23-038, TV-G-24-033, TV-G-24-094) or France (CREMEAS, APAFIS n°2020042818477700), and conducted in accordance with EU Directive 2010/63/EU. Prior power calculations for behavioral assessments were performed to minimize the number of animals used.<br>Rats: All pharmacokinetic studies were conducted in rats at Aptuit (Verona, Italy). All experimental procedures complied with the highest standards of animal welfare and were performed in accordance with the Italian Legislative Decree No. 26/2014 and European Directive No. 2010/63/EU. The studies were approved by the internal Aptuit Committee on Animal Research and Ethics and authorized by the Italian Ministry of Health (Project Authorization Code No. 35222). General procedures for animal care and housing followed the current recommendations of the Association for Assessment and Accreditation of Laboratory Animal Care (AAALAC).

Note that full information on the approval of the study protocol must also be provided in the manuscript.

Plants

Seed stocks	NA
Novel plant genotypes	NA
Authentication	NA
